# Global impact of World Sepsis Day on digital awareness of sepsis: an evaluation using Google Trends

**DOI:** 10.1186/s13054-018-1981-5

**Published:** 2018-03-09

**Authors:** Jelmer Savelkoel, Theodora A. M. Claushuis, Tjitske S. R. van Engelen, Luuk J. J. Scheres, W. Joost Wiersinga

**Affiliations:** 1 Department of Medicine and Division of Infectious Disease, Academic Medical Center, University of Amsterdam, Meibergdreef 9, Room G2-130, 1105 AZ Amsterdam, The Netherlands; 2 Center for Experimental Molecular Medicine, Academic Medical Center, University of Amsterdam, Meibergdreef 9, Room F0-117, 1105 AZ Amsterdam, The Netherlands; 3 Department of Vascular Medicine, Academic Medical Center, University of Amsterdam, Meibergdreef 9, 1105 AZ Amsterdam, The Netherlands; 4 Department of Clinical Epidemiology, Leiden University Medical Center, Leiden, The Netherlands

World Sepsis Day (WSD) was established by the Global Sepsis Alliance in 2012 and is held every 13^th^ of September. One of the objectives is to raise global awareness of sepsis. Despite its high mortality rate [1], an international survey reported that 80–90% of the public in western countries are unfamiliar with sepsis [2]. Anno 2018, public knowledge is no longer solely obtained via television and newspapers, but is largely acquired via the Internet and social media. These resources therefore contribute to digital awareness, and can be used to share knowledge. We aimed to investigate whether WSD is indeed associated with a global increase in digital information-seeking behaviour.

By using Google Trends™ data, which are presented as the relative search volume (RSV) [3], we investigated global digital information-seeking on the terms “sepsis”, “septicemia” and “blood poisoning”. The methods were similar to previous work that investigated the effect of World Thrombosis Day on digital information-seeking [4]. The years 2012–2016, in which WSD was held, were considered as exposure years, with the preceding 5 years (i.e. 2007–2011) serving as control years. The period of interest was defined as the 4 weeks surrounding WSD and compared with the control period, defined as the remaining weeks of the year. Global RSV data were downloaded on the 29^th^ of September 2017 using the “health” category. Data were downloaded for each year separately. Mean differences in RSV, both absolute and as percentages, between the period of interest and the control period were estimated for each year separately.

In the years that WSD was held, with the exception of the year 2012 when WSD was first introduced, we found a significant increase in digital information-seeking for the weeks surrounding WSD on terms related to sepsis compared with the remaining weeks of the year (Table 1 and Fig. 1). This was not the case for the years in which WSD was not yet held. The strengths of our approach are the focus on all-encompassing terms and the ability of comparing exposure years to control years. However, we assumed that an increase in digital information-seeking reflects an increase in awareness on sepsis, but we do not know whether an increase in digital information-seeking equals an increase in awareness.

**Table 1:** Mean differences in relative search volume between the period of interest and the control period

Year	Mean RSV in the 4 weeks surrounding WSD	Mean RSV in the remaining weeks of the year	Mean difference in RSV (95% CI)	*P* value
2007	59.8	54.7	5.1 (−3.0; 13.2)	0.215
2008	81.8	80.1	1.6 (−7.0; 10.3)	0.707
2009	50.3	49.6	0.6 (−10.0; 11.3)	0.904
2010	61.8	61.3	0.5 (−7.8; 8.8)	0.908
2011	77.3	75.4	1.9 (−5.4; 9.2)	0.608
2012 (WSD)	84.0	72.9	11.1 (−6.6; 28.7)	0.142
2013 (WSD)	84.0	72.5	11.5 (5.1; 17.9)	0.001
2014 (WSD)	92.0	81.8	10.3 (4.0; 16.5)	0.002
2015 (WSD)	94.0	82.5	11.5 (5.6; 17.3)	0.000
2016 (WSD)	64.5	51.7	12.8 (2.1; 23.6)	0.021

Mean difference in RSV between the period of interest (4 weeks surrounding WSD) and the control period (remaining weeks of the corresponding year) provided with the 95% confidence interval and *P* value

*P* values are based on the two-tailed *t* test for computing the statistical significance. *P* < 0.05 was considered significant

*WSD* World Sepsis Day, *RSV* relative search volume, *CI* confidence interval

**Fig. 1:**
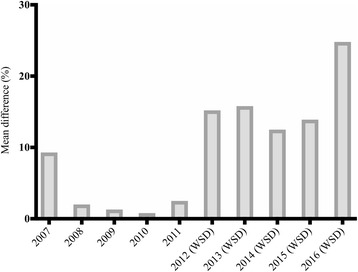
Mean differences in relative search volume between the period of interest (4 weeks surrounding World Sepsis Day) and the control period (remaining weeks of the corresponding year) expressed as percentages. *WSD* World Sepsis Day

In conclusion, our findings suggest that WSD has an important impact on digital awareness, which could be objectified with Google Trends™.

## Abbreviations

RSV: Relative search volume
WSD: World Sepsis Day
